# Could Obesity be a Triggering Factor for Endometrial Tubal Metaplasia to be a Precancerous Lesion?

**DOI:** 10.1155/2020/2825905

**Published:** 2020-03-20

**Authors:** Ayman M. El-Saka, Yomna A. Zamzam, Yosra A. Zamzam, Ayman El-Dorf

**Affiliations:** ^1^Department of Pathology, Faculty of Medicine, Tanta University, Tanta, Egypt; ^2^Department of Clinical Pathology, Faculty of Medicine, Tanta University, Tanta, Egypt; ^3^Department of Gynecology and Obstetrics, Faculty of Medicine, Tanta University, Tanta, Egypt

## Abstract

**Background & Aims:**

Endometrial tubal metaplasia (ETM) is mostly described in conjunction with unopposed estrogen levels, and its association with endometrial hyperplasia and endometrial carcinoma (EC) is striking. Obesity is a risk factor for endometrial hyperplasia and EC development. The aim of this study is to investigate the impact of BMI and serum estradiol level on expression of PAX-2, H-TERT, P16, Ki-67, and P53 in studied ETM in reference to benign endometrium and EC.

**Methods:**

The study was conducted on the following groups: group (1) consists of 57 cases that had endometrial biopsies with histologically demonstrable ETM (typical or atypical) and all were subjected to serum estradiol levelling and body mass index (BMI) evaluation; group (2) had adjacent benign endometrial tissue as control; group (3) consists of 52 cases of conventional endometrial carcinoma and 16 serous carcinoma paraffin blocks which were collected and reevaluated. All included groups were immunostained for PAX-2, H-TERT, p16, ki67, and p53.

**Results:**

The relation between BMI and serum estradiol level in group 1 and PAX-2, H-TERT, P16, and p53 was statistically significant, while their relation with atypia and ki67 expression was insignificant. Twenty-three ETM cases (40.4%) out of group 1 were all (100%) obese, 87% had high serum estradiol level, and 73.9% were postmenopausal and had a similar immunohistochemical profile as EC cases (group 3).

**Conclusions:**

The presence of ETM regardless of the histologic atypia in obese postmenopausal patients with high serum estradiol level is an alarming sign. This implies that ETM might not be as benign as generally accepted, as under certain clinical conditions, it may turn into a potential premalignant lesion.

## 1. Introduction

Tubal (or ciliated cell) metaplasia of the endometrium is characterized by ciliated columnar cells with bland round nuclei and eosinophilic cytoplasm, similar to the cells normally seen lining the fallopian tube [1]. It is generally thought that endometrial tubal metaplasia (ETM) is a benign disease. However, some studies propose ETM to be a potential premalignant endometrial lesion and has an association with endometrial hyperplasia, endometrial intraepithelial neoplasia (EIN), and well-differentiated endometrial carcinoma (EC) [2].

Even though ETM does not always progress to neoplasia, a determined effort should be performed on its presence in endometrial samples to detect any coexisting neoplastic condition [3].

Obesity is thought to cause an increase in EC risk due to abnormal levels of several hormones, most notably increased estrogen levels. Estrogen is a known endometrial growth factor, and, after menopause, the primary source of estrogen is peripheral tissues, including adipose tissue [4]. Bessonova et al. [5] reported a significant increase in risk of developing endometrial cancer for each 5 kg/m^2^ increase in body mass index (BMI) in women. Accordingly, postmenopausal obese women had an increase of both circulating estrone (E1) and estradiol (E2) fortyfold as compared to postmenopausal women with a normal BMI range [6].

PAX2 expression was described as a marker of the Mullerian duct derivatives as fallopian tubes, uterus, cervix, and upper vagina [7]. According to many studies, normal endometrial glandular cells have nuclear expression of PAX2 as revealed by immunohistochemistry. On the other hand, PAX2 expression was absent in EC and EIN implicating PAX2 as a possible key regulator in endometrial carcinogenesis [8, 9].

Human telomerase reverse transcriptase (H-TERT) is a catalytic subunit with reverse transcriptase activity. hTERC is constitutively present in normal and cancer cells, whereas expression of H-TERT is almost exclusively limited to cancer cells [10].

Scattered patterns or focal expression of p16 positive cells in serous EC and tubal tissue was described by several authors. They describe a pattern which is not seen in normal tissue and is different from diffuse p16 positivity as seen in high-risk HPV positive cervical intraepithelial lesions [11]. Inoue [12] reported that endometrial metaplasia, in general, was a precursor of variant types of endometrial carcinomas, based on the p53, PCNA, and ki67 overexpression in EC and endometrial metaplasia and the fact that EC are often accompanied by adjacent metaplastic epithelium [13]. However, it is, for now, not a generally accepted view that endometrial metaplasia is a precursor of endometrial cancer.

The current study was designed to investigate the impact of BMI and serum estradiol level on expression of PAX-2, H-TERT, P16, Ki-67, and P53 in studied ETM in reference to benign endometrium and EC.

## 2. Patients and Methods

### 2.1. Case Selection

The study was conducted on three groups. In group (1), from June 2016 to June 2019, fifty-seven cases of benign endometrial biopsies with histologically demonstrable tubal metaplasia (typical or atypical) were identified, reviewed, and included in this study. These samples were obtained from uterine curettage of 57 women aged from 28 to 72 years who were admitted to Gynecology and Obstetrics Department of Tanta University Hospitals complaining from dysfunctional uterine bleeding. After histological diagnosis of tubal metaplasia, all 57 patients were subjected to serum estradiol levelling and body mass index (BMI) evaluation. In group (2), the adjacent benign endometrial tissue was used as control. In group (3), for further comparison, Tanta Pathology Department archives were searched from January 2016 through to March 2019 and sixty-eight previously diagnosed EC (52 cases with conventional endometrial carcinoma and 16 cases with serous carcinoma) and study paraffin blocks were collected and reevaluated. Any cases of EC with adjacent ETM were excluded from this study.

This study was approved by the ethics committee of Faculty of Medicine, Tanta University. All included patients provided a written informed consent for the use of biological specimens for research purposes.

### 2.2. Histopathological Evaluation

All included 57 tissue biopsies (groups 1 and 2) were fixed in formalin, embedded as paraffin blocks and cut at 4 *μ*m, and stained with hematoxylin and eosin (H & E). After histopathological evaluation by two separate pathologists, they were diagnosed as follows: tubal metaplasia (nonmetaplastic endometrium having some ciliated cells and resembling fallopian tube) with adjacent 22 cases with disordered proliferative endometrium, 5 cases with atrophic endometrium, 6 cases with senile cystic fibrosis, and 24 cases with benign endometrial hyperplasia (used as control). Atypical tubal metaplasia was found in 24 cases. The criteria for cytologic atypia of atypical tubal metaplasia included pleomorphic and hyperchromatic nuclei and increased nuclear-to-cytoplasmic ratio. Atypical endometrial hyperplasia or EC was not identified in any studied cases.

The collected 68 paraffin blocks (group 3) were also recut and stained with H and E, reevaluated, and diagnosed as 52 cases with conventional endometrial carcinoma and 16 cases with serous carcinoma.

### 2.3. Immunohistochemical Analysis

All included groups were immunostained for PAX-2, H-TERT, p16, ki67, and p53 (Table 1). Briefly, all tissues were deparaffinized followed by blockade of endogenous peroxidases and antigen retrieval using Antigen Unmasking Solution (Vector; USA), followed by the primary antibody and then the visualization reagent (secondary goat anti-mouse immunoglobulin and horseradish peroxidase linked to a dextran polymer backbone). After rinsing with distilled water, the slides were incubated with DAB (3, 3-diaminobenzidine) substrate–chromagen solution and counterstained with hematoxylin. Negative controls were prepared using primary antibody with PBS and normal mouse or rabbit serum.

#### 2.3.1. Staining Scoring

Immunochemical results were independently scored by two experienced pathologists who were blinded to the patients' clinicopathological data and outcomes. The pathologists reviewed immunostained tissue sections under a light microscope. The ranges used for PAX-2 expression analysis were as follows: 0%, 1–25%, 26–50%, 51–75%, or 76–100%. Positive PAX-2 expression was only considered with a nuclear staining pattern. PAX-2 loss was scored as follows: (1) complete loss (0% cells staining), (2) partial loss (1–75% cells staining), and (3) minimal to no loss (76–100% cells staining) [9].

The H-TERT immunostaining score was calculated with a semiquantitative scoring system according to Qin et al. [14] (only diffuse nuclear was considered as positive). The intensity range was as follows: 0, no staining; 1, weak staining; 2, moderate staining; 3, strong staining. The extent of staining was presented as percentage of tumor cells that were stained: 0, <10% of tumor cells stained; 1, 10–50% of positive cells; 2, >50 and <75% of positive cells; 3, >75% of positive cells. An overall score was obtained as the product of the intensity and the extent of positive staining. Cases with 0 points were considered to be negative (0), cases with a score of 1–3 weakly positive (1+), cases with a score of 4–7 moderately positive (2+), and cases with a score of >7 strongly positive (3+).

For p16, the staining was scored as diffuse (>80%) strong (2), focal (5% to 80%) strong (1), or negative (<5%) based on the nuclear and cytoplasmic staining for p16 [11].

For ki67 and p53 staining assessment, nuclear staining intensity was scored using three categories; mild, moderate, and strong. The staining ratio was scored as 0 for no staining, 1 for <10%, 2 for 10% to 50%, and 3 for >50% [13].

#### 2.3.2. Enzyme-Linked Immunosorbent Assay (ELISA) Evaluation of Serum Estradiol and BMI Evaluation

All group 1 patients were subjected to serum estradiol levels analysis. A peripheral blood sample (10 ml) was collected in sterile tubes. After clotting the serum for 30 minutes at room temperature in a serum separator tube, it was centrifuged for 15 min and then analyzed immediately and frozen at −20°C for storage. Serum estradiol levels were quantified by an estradiol enzyme-linked immunosorbent assay (ELISA) kit as recommended by the instructions of the manufacturer (R&D Systems, Minneapolis, MN, USA) with normal range 50–300 ng/ml. Serum estradiol level of more than 300 ng/ml was considered high [15].

BMI was calculated using the following equation: weight (kg)/height (m^2^). WHO defines being underweight as 18.5, normal weight as 18.5–24, overweight as 25–29.9, and obese ≥30 (kg/m^2^). In this study, the patients were classified as nonobese <30 and obese ≥30 (kg/m^2^) [16].

#### 2.3.3. Statistical Analysis

Statistical presentation and analysis of the present study were conducted, using the mean, standard deviation, and chi-square test, Student's *t*-test, and Mann–Whitney test by SPSS software (version 15.0; SPSS Inc., Chicago, Illinois, USA). Significant differences were considered as *p* < 0.05.

## 3. Results

The clinicopathological characteristics of the three studied groups are summarized in Table 2. Group 1 included 57 tubal metaplasia patients, group 2 included 57 adjacent benign endometrial samples as control, and group 3 included 68 endometrial carcinoma cases. In group 1, the age of the included patients ranged from 28 to 72 years with a mean age of 49.3 ± 13.6 years. Out of the studied 57 patients, 32 (56.1%) were premenopausal and 25 (43.9%) were postmenopausal. Regarding history of oral contraceptive intake, 28 patients (49.1%) were positive and 29 patients (50.9%) were negative. Twenty-five patients (43.9%) had histological atypia on microscopic examination of their endometrial biopsy while 32 (56.1%) showed no histologic atypia. According to BMI, 23 patients (40.4%) were obese and 34 patients (59.6%) were nonobese. Serum estradiol level was high (>300 ng/ml) in 20 patients (35.1%) although 37 patients (64.9%) had a level of ≤300 ng/ml. In group 2, according to histologic typing, disordered proliferative endometrium was found in 22 (38.6%) samples and atrophic endometrium in 5 (8.8%)

Senile cystic fibrosis was found in 6 (10.5%) samples and benign endometrial hyperplasia in 24 (42.1%). In group 3, regarding histologic typing, serous endometrial carcinoma was rediagnosed in 16 (23.5%) cases and conventional endometrial carcinoma diagnosis in 52 (76.5%).

**Figure 1:**
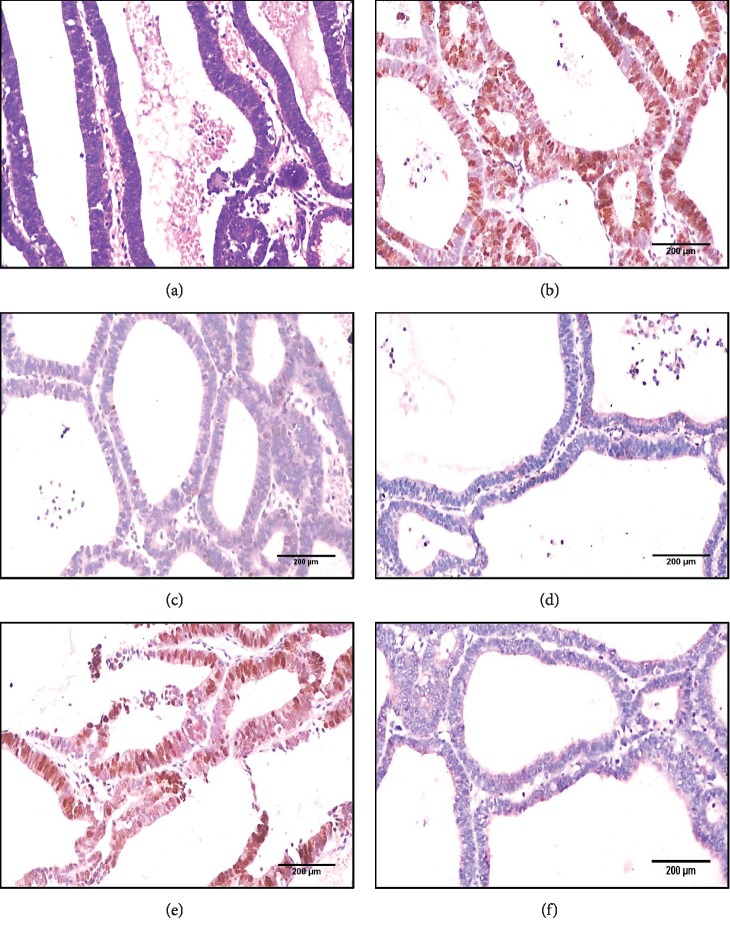
(a) An immunohistochemistry panel for ETM case (case with BMI 22 kg/m^2^ and serum estradiol level 180 ng/ml showing glands lined with tubal-like epithelium exhibiting atypical changes pleomorphism, hyperchromasia, and high N/C ratio (H&E X 100)), (b) PAX2 strong positive nuclear expression score 3 (X 100), (c) H-TERT negative nuclear expression (X 100), (d) P16 negative expression (X 100), (e) Ki67 strong nuclear expression score 3 (X 100), and (f) P53 negative expression (X 100).

**Figure 2:**
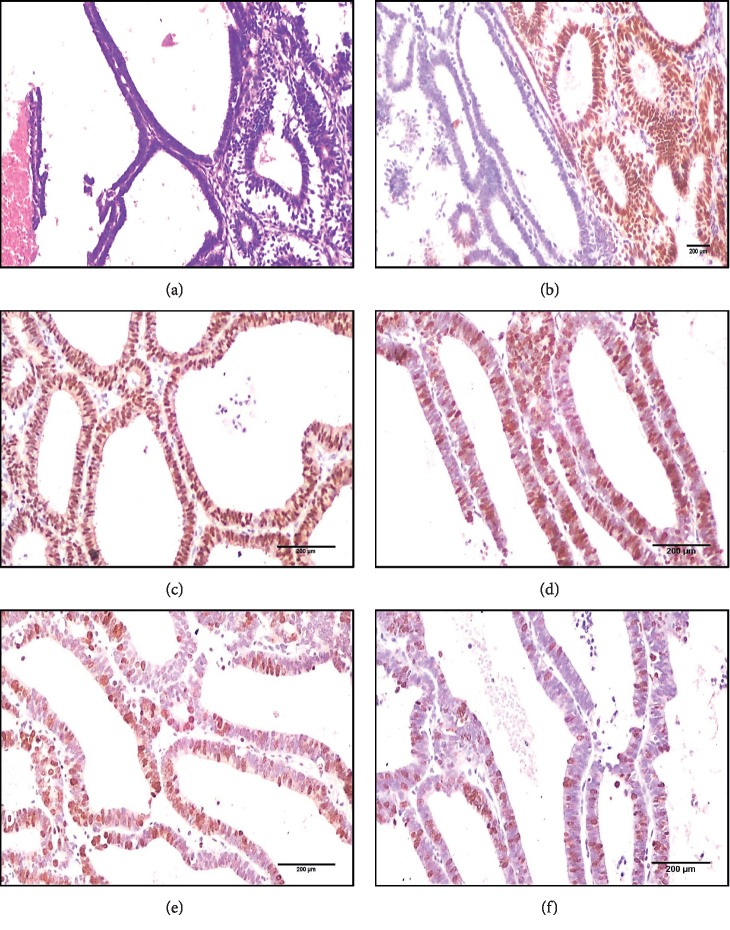
(a) An immunohistochemistry panel for ETM case (a case with BMI 38 kg/m^2^ and serum estradiol level 420 ng/ml showing glands lined with tubal-like epithelium with adjacent benign endometrial glands without metaplasia (H&E X 40)), (b) PAX2 complete negative expression (score 1) in glands with tubal metaplasia and positive nuclear expression in the adjacent nonmetaplastic glands (X 40), (c) H-TERT strong diffuse positive nuclear expression score 3 (X 100), (d) P16 focal strong nuclear and cytoplasmic expression score 1 (X 100), (e) Ki67 strong nuclear expression score 3 (X 100), and (f) P53 focal strong nuclear expression score 2 (X 100).

**Figure 3:**
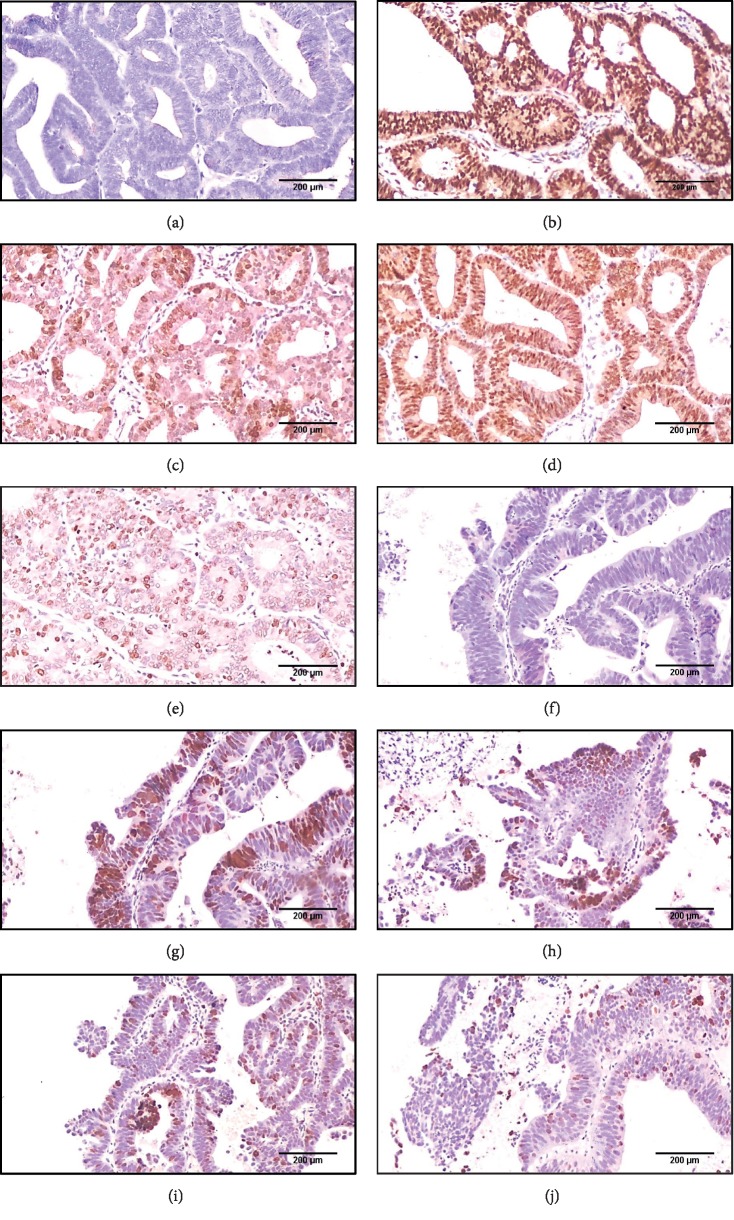
An immunohistochemistry panel for conventional EC: (a) PAX2 complete negative expression, score 1 (X 100), (b) H-TERT strong diffuse positive nuclear expression score 3 (X 100), (c) P16 focal strong nuclear and cytoplasmic expression score 1 (X 100), (d) Ki67 diffuse strong nuclear expression score 3 (X 100), and (e) P53 focal strong nuclear expression score 2 (X 100). An immunohistochemistry panel for papillary serous EC: (f) PAX2 complete negative expression, score 1 (X 100), (g) H-TERT strong positive nuclear expression score 3 (X 100), (h) P16 focal strong nuclear and cytoplasmic expression score 1 (X 100), (i) Ki67 strong nuclear expression score 2 (X 100), and (j) P53 focal strong nuclear expression score 2 (X 100).

**Figure 4:**
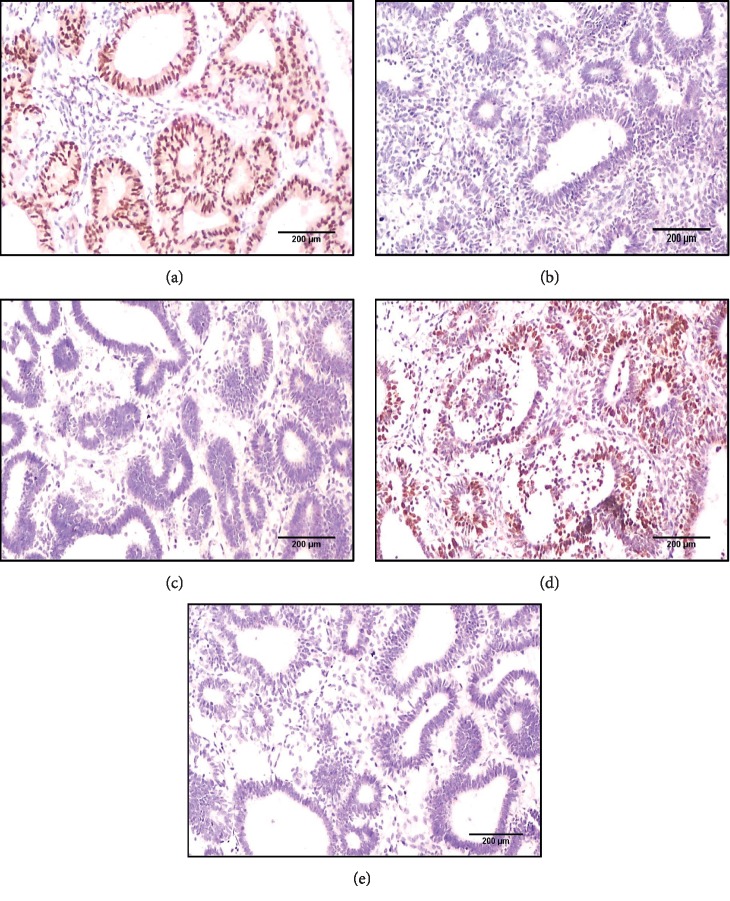
(a) An immunohistochemistry panel for benign endometrial hyperplasia case: PAX2 strong positive nuclear expression score 3 (X 100), (b) H-TERT negative nuclear expression (X100), (c) P16 negative expression (X 100), (d) Ki67 strong nuclear expression score 3 (X 100), and (e) P53 negative expression (X 100).

There was a significant relationship between studied markers in the three different groups as illustrated in Table 3. PAX2 expression was negative in 23 cases (40.4%) out of group 1, 5 cases (8.8%) of group 2, and 57 cases (83.8%) of group 3. H-TERT expression was positive in 21 cases (36.8%) out of group 1, four cases (7%) of group 2, and 58 cases (85.3%) of group 3. P16 expression was positive in 23 cases (40.4%) out of group 1, seven cases (12.3%) of group 2, and 23 cases (33.8%) of group 3 especially in serous carcinoma type. Ki67 expression was positive in 55 cases (96.5%) out of group 1, 47 cases (82.5%) of group 2, and in all cases of group 3 (68 cases, 100%). P53 expression was positive in 26 cases (45.6%) out of group 1, 11 cases (19.3%) of group 2, and 33 cases (48.5%) of group 3 (Figures 1–4).

The relation between BMI, age, menopausal status, serum estradiol level, PAX-2, H-TERT, P16, and p53 expression was statistically significant. On the other hand, the relation between BMI, history of oral contraceptive pills, presence of histologic atypia, and ki67 expression was statistically insignificant as shown in Table 4. Out of the 23 included obese patients (≥30 kg/m^2^), 17 patients (73.9%) were postmenopausal, 20 patients (87%) had serum estradiol level of more than 300 ng/ml, 21 obtained endometrial samples (91.3%) were negative for PAX-2 expression, 20 (87%) samples were positive for both H-TERT and p53 expression, and 18 samples (78.3%) were positive for p16 expression.

Regarding serum estradiol level, there was a statistically significant relationship with age, menopausal status, PAX-2, H-TERT, and P16 and p53 expression, while the relation between serum estradiol level, history of oral contraceptive pills, presence of histologic atypia, and ki67 expression was statistically insignificant as illustrated in Table 5. Out of the 20 included patients (>300 ng/ml), 14 patients (70%) were postmenopausal, 18 obtained endometrial samples (90%) were negative for PAX-2 expression, 17 (85%) samples were positive for H-TERT, 16 samples (80%) were positive for p16 expression, and 18 samples (90%) were positive for p53 expression.

Histologic atypia in studied cases of group 1 was statistically insignificant with all other clinicopathological characters and studied markers expression as described in Table 6.

## 4. Discussion

ETM is mostly described in conjunction with unopposed estrogen levels, and its association with simple and complex endometrial hyperplasia and well-differentiated EC is striking [17]. Some authors suggested that the presence of atypical endometrial hyperplasia is an alarming sign for suspecting of malignant transformation [11, 18]. On the other hand, other authors thought that the presence of architecturally complex glands in ETM, even with no atypia, is often associated with endometrioid adenocarcinoma and consequently should be managed as a case of complex endometrial hyperplasia [19, 20].

To our knowledge this the first study to assess the impact of BMI and serum estradiol level on ETM with or without presence of atypia and their role as precursor of EC. The current study investigates the impact of BMI and serum estradiol level on expression of PAX-2, H-TERT, P16, Ki-67, and P53 in studied ETM in reference to benign endometrium and EC.

In the present study, PAX2 expression was absent in 23 cases (40.4%) out of group 1, 5 cases (8.8%) of group 2, and 57 cases (83.8%) of group 3. This relation was statistically significant. Also, the relation between PAX-2 expression and BMI and serum estradiol level in group 1 was statistically significant, while its relation with atypia was insignificant (*p*=0.962). Our findings are similar to those of Cao et al. [8] and Allison et al. [9] in terms of PAX2 expression in benign endometrial lesions and EC. They found that PAX2 protein expression had progressive loss along the spectrum from hyperplasia to EC, but unfortunately, they did not include ETM cases in their studies.

Wu et al. [21] described PAX2 expression in endometrial cancer cell lines. They stated that tamoxifen and estrogen could activate PAX2 mRNA expression in endometrial cancer cell lines but not in normal endometrial samples. This increased expression was associated with cancer-linked hypomethylation of the PAX2 promoter. These results appeared to offer a mechanism to explain the increased incidence of endometrial cancers in women with unopposed estrogen in obese patients with high serum estradiol level especially in the postmenopausal state.

In the current study, H-TERT expression was positive in 21 cases (36.8%) out of group 1, 4 cases (7%) of group 2, and 58 cases (85.3%) of group 3. This relation was statistically significant. Also, the relation between H-TERT expression and BMI and serum estradiol level in group 1 was statistically significant, while its relation with atypia was insignificant (*p*=0.503). Some researches were in accordance with these results which stated that H-TERT expression was limited almost exclusively to cancer cells reporting that the immunohistochemical reactivity of TERT was present in numerous human cancers, including uterine endometrioid and serous carcinoma, but not in benign lesions such as disordered proliferative endometrium and benign endometrial hyperplasia [10, 22]. On the other hand, Simon et al. [3] reported that H-TERT immunopositivity was present in uterine serous carcinoma, and in contrast, atypical tubal metaplasia was completely negative for TERT. This could be attributed to a smaller number of ETM cases (only 16) who were all atypical. They did not include other cases of ETM without atypia in their study.

In this study, P16 expression was positive in 23 cases (40.4%) out of group 1, seven cases (12.3%) of group 2, and 23 cases (33.8%) of group 3. This relation was statistically significant. Also, the relation between p16 expression with BMI and serum estradiol level in group 1 was statistically significant, while its relation with atypia was insignificant (*p*= 0.620). Horree et al. [11] proposed that TM of the endometrium could be a potentially premalignant lesion. They explained their finding that expression of p16 is regarded as a carcinogenetic event in many tumors, including those in the gynecological tract [23, 24]. Further, Umezaki [25] suggested that tubal metaplasia should be considered as a neoplastic lesion of uterine cervical glandular lesions that may have the potential to undergo malignant transformation. p16 is also aberrantly expressed in TM in some ovarian inclusion cysts. These cysts have been proposed to be precursors of ovarian cancer [26].

For Ki67 expression, it was positive in 55 cases (96.5%) out of group 1, 47 cases (82.5%) out of group 2, and in all cases of group 3 (68 cases, 100%). This relation was statistically significant. However, the relation between ki67 expression with BMI, serum estradiol level, and histologic atypia in group 1 was statistically insignificant. It is well known that Ki-67 is expressed exclusively in proliferating cells. Studies have also shown that Ki-67 proliferation indices are increased in numerous human malignancies, including high-grade EC as well as atypical endometrial hyperplasia, as compared with benign endometrial lesions [18, 27]. On contrary to our results, Simon et al. [3] reported that the majority of atypical tubal metaplasia either is negative for Ki-67 or shows a low proliferation index similar to that seen in typical tubal metaplasia, and this could be explained by their small sample size.

For P53 expression, it was positive in 26 cases (45.6%) out of group 1, 11 cases (19.3%) out of group 2, and 33 cases (48.5%) out of group 3. This relation was statistically significant. Besides, the relation between p53 expression with BMI and serum estradiol level in group 1 was statistically significant, while its relation with atypia was insignificant (*p*=0.829). This was in agreement with Simon et al. [3] who showed that atypical tubal metaplasia displayed negative or focal, weak immunoreactivity with p53. The presence of weak and heterogeneous p53 immunoreactivity in ETM could be a consequence of DNA damage. While intense, diffuse and homogeneous p53 staining favors carcinoma [1].

In the present study, it was found that 23 ETM cases (40.4%) out of group 1 were all (100%) obese (more than 30 kg/m^2^), 87% had high serum estradiol level of more than 300 ng/ml, and 73.9% were postmenopausal and had a similar immunohistochemical profile as EC cases (group 3). This suggests that certain clinical conditions could be triggering factors for ETM to be a precursor lesion for EC. Cases of ETM, especially those with certain previous conditions, need clinical followup in the form of regular uterine ultrasound examination of endometrial thickness and subendometrial vascularity every three months at least.

## 5. Conclusions

The presence of ETM regardless of the histologic atypia in obese postmenopausal patients with high serum estradiol level is an alarming sign for patients strict followup. This implies that ETM might not be as benign as generally accepted, as under certain clinical conditions, it may turn into a potential premalignant lesion. After all, studies on a larger scale should be performed to validate the precancerous potential of ETM under those certain conditions.

## Figures and Tables

**Table 1:** Antibodies used in this study.

Antibody to	Clone	Company	Raised in	Positive control	Antigen retrieval method	Dilution and incubation time	Localization
PAX-2	Z-RX2 polyclonal	Invitrogen, Camarillo, CA	Rabbit	Renal cell carcinoma	Citrate buffer, pH 9.0	1 : 300, overnight, 4°C	Nuclear

H-TERT	sc-7215, code: c20 monoclonal	Santa Cruz Biotechnology, Dallas, TX, USA	Goat	Squamous cell carcinoma	Citrate buffer pH 6.0	1 : 50, overnight, 4°C	Nuclear

P16	SC-468 polyclonal	Santa Cruz Biotechnology, Santa Cruz, CA	Rabbit	Uterine cervical sections	Citrate buffer ph 6.0	1 : 250, overnight, 4°C	Cytoplasmic and nuclear

Ki67	Mib-1 monoclonal	DAKO, Glostrup, Denmark	Mouse	Lymph node germinal center	Citrate buffer ph 6.0	1 : 100, overnight, 4°C	Nuclear

P53	DO-7 monoclonal	DAKO, Glostrup, Denmark	Mouse	Colon carcinoma	Citrate buffer pH 6.0	1 : 100, overnight, 4°C	Nuclear

**Table 2:** Distribution of the studied cases according to different parameters in all studied groups.

Parameter	No. (%)
*Group 1 (n* *=* *57)*	

Age (years)	
Mean ± SD	49.3 ± 13.6
Median (min–max.)	43 (28–72)
Menopausal status	
Premenopausal	32 (56.1%)
Postmenopausal	25 (43.9%)
Oral contraceptive history	
Negative	29 (50.9%)
Positive	28 (49.1%)
Atypia	
No atypia	32 (56.1%)
Atypia	25 (43.9%)
BMI (kg/m^2^)	
Nonobese (<30)	34 (59.6%)
Obese (≥30)	23 (40.4%)
Mean ± SD	30.5 ± 7.4
Median (min–max.)	28 (18–45)
Estradiol level	
≤300	37 (64.9%)
>300	20 (35.1%)
Mean ± SD	247.8 ± 124.6
Median (min–max.)	240 (60–450)

*Group 2 (n* *=* *57)*	

Histologic type	
Disordered proliferative endometrium	22 (38.6%)
Atrophic endometrium	5 (8.8%)
Senile cystic fibrosis	6 (10.5%)
Benign endometrial hyperplasia	24 (42.1%)

*Group 3 (n* *=* *68)*	

Histologic type	
Serous	16 (23.5%)
Conventional	52 (76.5%)

**Table 3:** Comparison between the three studied groups according to study markers.

	Group 1 (*n* = 57)	Group 2 (*n* = 57)	Group 3 (*n* = 68)	*χ* ^2^	*p*
PAX-2					
Negative	23 (40.4%)	5 (8.8%)	57 (83.8%)	71.515^*∗*^	<0.001^*∗*^
Positive	34 (59.6%)	52 (91.2%)	11 (16.2%)		

PAX-2 loss score					
1	23 (40.4%)	5 (8.8%)	31 (45.6%)	27.376^*∗*^	<0.001^*∗*^
2	15 (26.3%)	24 (42.1%)	26 (38.2%)		
3	19 (33.3%)	28 (49.1%)	11 (16.2%)		

H-TERT					
Negative	36 (63.2%)	53 (93%)	10 (14.7%)	79.158^*∗*^	<0.001^*∗*^
Positive	21 (36.8%)	4 (7%)	58 (85.3%)		

H-TERT score					
0	36 (63.2%)	53 (93%)	10 (14.7%)	103.941^*∗*^	<0.001^*∗*^
1	1 (1.8%)	4 (7%)	3 (4.4%)		
2	12 (21.1%)	0 (0%)	25 (36.8%)		
3	8 (14%)	0 (0%)	30 (44.1%)		

P16					
Negative	34 (59.6%)	50 (87.7%)	45 (66.2%)	12.043^*∗*^	0.002^*∗*^
Positive	23 (40.4%)	7 (12.3%)	23 (33.8%)		

P16 score					
0	34 (59.6%)	50 (87.7%)	46 (67.6%)	19.369^*∗*^	0.001^*∗*^
1	8 (14%)	7 (12.3%)	13 (19.1%)		
2	15 (26.3%)	0 (0%)	9 (13.2%)		

Ki67					
Negative	2 (3.5%)	10 (17.5%)	0 (0%)	15.250^*∗*^	<0.001^*∗*^
Positive	55 (96.5%)	47 (82.5%)	68 (100%)		

ki67 score					
0	2 (3.5%)	10 (17.5%)	0 (0%)	123.287^*∗*^	<0.001^*∗*^
1	16 (28.1%)	34 (59.6%)	0 (0%)		
2	17 (29.8%)	13 (22.8%)	16 (23.5%)		
3	22 (38.6%)	0 (0%)	52 (76.5%)		

P53					
Negative	31 (54.4%)	46 (80.7%)	35 (51.5%)	13.529^*∗*^	0.001^*∗*^
Positive	26 (45.6%)	11 (19.3%)	33 (48.5%)		

P53 score					
0	31 (54.4%)	46 (80.7%)	35 (51.5%)	25.634^*∗*^	<0.001^*∗*^
1	6 (10.5%)	11 (19.3%)	13 (19.1%)		
2	12 (21.1%)	0 (0%)	13 (19.1%)		
3	8 (14%)	0 (0%)	7 (10.3%)		

*χ* ^2^: chi-square test. *p*: *p* value for comparing between the studied groups. ^*∗*^ Statistically significant at *p* ≤  0.05.

**Table 4:** Relation between BMI and other parameters in group 1 (*n* = 57).

	BMI		Test of sig.	*p*
	Nonobese (<30) (*n* = 34)	Obese (≥30) (*n* = 23)		
Age (years)				
Mean ± SD	43.1 ± 10.7	58.5 ± 12.2	*t* = 5.038^*∗*^	<0.001^*∗*^
Median (min.–max.)	38.5 (28–68)	64 (37–72)		

Menopausal status				
Premenopausal	26 (76.5%)	6 (26.1%)	*χ* ^2^ = 14.144^*∗*^	<0.001^*∗*^
Postmenopausal	8 (23.5%)	17 (73.9%)		

Oral contraceptive history				
Negative	21 (61.8%)	8 (34.8%)	*χ* ^2^ = 3.996^*∗*^	0.046^*∗*^
Positive	13 (38.2%)	15 (65.2%)		

Atypia				
No atypia	18 (52.9%)	14 (60.9%)	*χ* ^2^ = 0.350	0.554
Atypia	16 (47.1%)	9 (39.1%)		

Serum estradiol level				
≤300	34 (100%)	3 (13%)	*χ* ^2^ = 45.546^*∗*^	<0.001^*∗*^
>300	0 (0%)	20 (87%)		
Mean ± SD	160.4 ± 62.6	377 ± 66.3	*U* = 2.0	<0.001^*∗*^
Median (min. –max.)	150 (60–290)	420 (280–450)		

PAX-2				
Negative	2 (5.9%)	21 (91.3%)	*χ* ^2^ = 41.592^*∗*^	<0.001^*∗*^
Positive	32 (94.1%)	2 (8.7%)		

H-TERT				
Negative	33 (97.1%)	3 (13%)	*χ* ^2^ = 41.618^*∗*^	<0.001^*∗*^
Positive	1 (2.9%)	20 (87%)		

P16				
Negative	29 (85.3%)	5 (21.7%)	*χ* ^2^ = 23.024^*∗*^	<0.001^*∗*^
Positive	5 (14.7%)	18 (78.3%)		

Ki67				
Negative	2 (5.9%)	0 (0%)	*χ* ^2^ = 1.402	0.510
Positive	32 (94.1%)	23 (100%)		

P53				
Negative	28 (82.4%)	3 (13%)	*χ* ^2^ = 26.566^*∗*^	<0.001^*∗*^
Positive	6 (17.6%)	20 (87%)		

*χ* ^2^: chi-square test; *t*: Student's *t*-test; U: Mann-Whitney test. *p*:*p* value for association between BMI and different markers. ^*∗*^Statistically significant at *p* ≤ 0.05.

**Table 5:** Relation between serum estradiol level and other parameters in group 1 (*n* = 57).

	Estradiol level		Test of sig.	*p*
	≤300 (*n* = 37)	>300 (*n* = 20)		
Age (years)				
Mean ± SD	44.6 ± 11.6	58 ± 13	*t* = 3.961^*∗*^	<0.001^*∗*^
Median (min.–max.)	39 (28–68)	64.5 (37–72)		

Menopausal status				
Premenopausal	26 (70.3%)	6 (30%)	*χ* ^2^ = 8.550^*∗*^	0.003^*∗*^
Postmenopausal	11 (29.7%)	14 (70%)		

Oral contraceptive history				
Negative	22 (59.5%)	7 (35%)	*χ* ^2^ = 3.108	0.078
Positive	15 (40.5%)	13 (65%)		

PAX-2				
Negative	5 (13.5%)	18 (90%)	*χ* ^2^ = 31.555^*∗*^	<0.001^*∗*^
Positive	32 (86.5%)	2 (10%)		

H-TERT				
Negative	33 (89.2%)	3 (15%)	*χ* ^2^ = 30.709^*∗*^	<0.001^*∗*^
Positive	4 (10.8%)	17 (85%)		

P16				
Negative	30 (81.1%)	4 (20%)	*χ* ^2^ = 20.124^*∗*^	<0.001^*∗*^
Positive	7 (18.9%)	16 (80%)		

Ki67				
Negative	2 (5.4%)	0 (0%)	*χ* ^2^ = 1.120	0.536
Positive	35 (94.6%)	20 (100%)		

P53				
Negative	29 (78.4%)	2 (10%)	*χ* ^2^ = 24.469^*∗*^	<0.001^*∗*^
Positive	8 (21.6%)	18 (90%)		

*χ* ^2^: chi-square test; *t*: Student's *t*-test. *p*: *p* value for association between atypia and different markers. ^*∗*^Statistically significant at *p* ≤ 0.05.

**Table 6:** Relation between histologic atypia and other parameters in group 1 (*n* = 57).

	Histologic atypia		Test of sig.	*p*
	No atypia (*n* = 32)	Atypia (*n* = 25)		
Age (years)				
Mean ± SD	51 ± 13.8	47.2 ± 13.3	*t* = 1.039	0.303
Median (min.–max.)	49.5 (28–72)	41 (33–72)		

Menopausal status				
Premenopausal	16 (50%)	16 (64%)	*χ* ^2^ = 1.117	0.291
Postmenopausal	16 (50%)	9 (36%)		

Oral contraceptive history				
Negative	16 (50%)	13 (52%)	*χ* ^2^ = 0.022	0.881
Positive	16 (50%)	12 (48%)		

PAX-2				
Negative	13 (40.6%)	10 (40%)	*χ* ^2^ = 0.002	0.962
Positive	19 (59.4%)	15 (60%)		

H-TERT				
Negative	19 (59.4%)	17 (68%)	*χ* ^2^ = 0.449	0.503
Positive	13 (40.6%)	8 (32%)		

P16				
Negative	20 (62.5%)	14 (56%)	*χ* ^2^ = 0.246	0.620
Positive	12 (37.5%)	11 (44%)		

Ki67				
Negative	2 (6.3%)	0 (0%)	*χ* ^2^ = 1.619	0.499
Positive	30 (93.8%)	25 (100%)		

P53				
Negative	17 (53.1%)	14 (56%)	*χ* ^2^ = 0.047	0.829
Positive	15 (46.9%)	11 (44%)		

*χ* ^2^: chi-square test; *t*: Student's *t*-test. *p*: *p* value for association between atypia and different markers. ^*∗*^Statistically significant at *p* ≤  0.05.

## Data Availability

The data supporting the results are present in the discussion and presented in the references section.
